# Body mass and sex, but not breeding condition and season, influence open‐field exploration in the yellow‐necked mouse

**DOI:** 10.1002/ece3.8771

**Published:** 2022-03-27

**Authors:** Paula A. Bednarz, Rafał Zwolak

**Affiliations:** ^1^ Department of Systematic Zoology Adam Mickiewicz University in Poznań Poznań Poland

**Keywords:** adults, asset‐protection principle, breeding condition, juveniles, subadults

## Abstract

Theory predicts that risk taking should be influenced by external (e.g., season) and internal (e.g., breeding condition, sex, and body mass) conditions. We investigated whether these factors are associated with a potentially risky behavior: exploration of a novel environment. We conducted repeated open‐field tests of exploration in a common forest rodent, the yellow‐necked mouse *Apodemus flavicollis*. Contrary to expectations, the exploration did not vary with the season (spring vs. fall) or the reproductive status of the tested animals. Also unexpectedly, there was an inverted U‐shaped relationship between body mass and exploration: animals with intermediate body mass tended to have the highest exploration tendencies. Males were more exploratory than females. Finally, even after adjusting for the effects of body mass and sex, individuals exhibited consistent, repeatable differences in exploration tendencies (“behavioral types” or “personalities”). The discrepancies between certain broad generalizations and our results suggest that risk taking depends on details of species‐specific biology.

## Graphical abstract

We placed wild rodents (yellow‐necked mice, *Apodemus flavicollis*) in a novel environment (an open arena) and evaluated how body mass, sex, reproductive status, and season affect their exploration. We found (1) an inverted U‐shaped relationship between body mass and exploration, (2) higher exploration in males than in females, and (3) no influence of season or reproductive status on exploration.

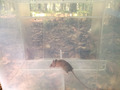

## 1 INTRODUCTION

Adequate behavioral responses to novel circumstances are a matter of life and death. Yet, there is no single “best” strategy for dealing with a new, potentially threatening situation. Instead, the optimal strategy should depend on internal (individual traits and state) and external (environmental) conditions. Thus, the propensity for risky behavior can depend on age (Macrì et al., 2002; Sherratt & Morand‐Ferron, 2018), sex (Apicella et al., 2017; Ensminger & Westneat, 2012), reproductive state (Bunnefeld et al., 2006), body condition (Moran et al., 2021), season (Eccard & Herde, 2013; Reinhardt & Healey, 1999), predation pressure (Berchtold & Côté, 2020), and so on. One of the most popular theories that frames this variation and predicts optimal behavior in the face of danger is the asset‐protection principle (Clark, 1994). The asset is reproductive value, defined as “expected future lifetime reproduction” (Fisher 1930 cited in Clark, 1994). Assuming that risky behavior brings more benefits (e.g., faster acquisition of resources), but increased risk of death relatively to cautious behavior, animals with higher reproductive value should be more cautious because they have more to lose. Therefore, optimal behavior should depend on factors that influence reproductive value (Clark, 1994).

Other explanations include age‐related shifts in exploration (Sherratt & Morand‐Ferron, 2018; Spreng & Turner, 2021) and sex‐specific selection for risk taking (Palanza, 2001a). The age‐related shifts result from the shorter time horizon that older individuals have to capitalize on the value of novel information, relative to younger ones. Thus, the benefit‐to‐cost ratio of exploration and the propensity to explore decline with age (Sherratt & Morand‐Ferron, 2018; Spreng & Turner, 2021). The sex‐specific selection states that sexes differ in cost and benefit of risky behavior. In mammals, risk taking is often more beneficial for males, who have relatively lower level of parental investment, and must compete with other males for matings, but can potentially sire numerous offspring (Davies et al., 2012; Palanza, 2001a). Females, the high investment sex, is expected to face less intense competition for mates, but is more limited in the number of potential progeny. The higher reproductive potential of males means that they have less to lose, and more to gain from risky behavior in comparison with females (Palanza, 2001a).

However, in addition to plastic responses that change, e.g., with reproductive value or age, asset protection can also create consistent among‐individual differences in behavior (a.k.a. “animal personalities” or “behavioral types”: Réale et al., 2007; Sih et al., 2004) through state‐behavior feedbacks. For example, being cautious can reduce current reproduction, but increase the chances of reproduction in the future. Conversely, risky behavior can increase current reproduction, but diminish chances of surviving to future reproductive events. In such a situation, asset protection can lead to emergence of fast (risky behavior and early reproduction) and slow (cautious behavior and delayed reproduction) lifestyles, where the fast ones are characterized by risky behavior and early reproduction, and the slow ones exhibit cautious behavior and delayed reproduction (Sih et al., 2015; Wolf et al., 2007). In this case, the plasticity of behavior would be constrained by the emergence of animal personalities (Sih et al., 2015). Yet, empirical support for the state‐behavior feedbacks is equivocal (Niemelä & Dingemanse, 2018).

The open‐field test is a classical tool for testing animal behavior. Originally developed to be used with rodents, it is now routinely applied to a variety of animal taxa (Perals et al., 2017). The test consists of placing an animal in an open arena (a novel environment) and quantifying its behavior over a fixed amount of time. The procedure is simple and allows rapid testing, but has been criticized for confounding different aspects of behavior (e.g., exploration, neophobia, and boldness), especially when various actions are measured (e.g., time to enter the arena, sniffing, defecations, rearing, and so on) (Carter et al., 2013). However, distance travelled in the open field (and its various indices) has been repeatedly demonstrated to be a reliable indicator of exploration in a novel environment (Finger et al., 2016; Lafaille & Féron, 2014; Perals et al., 2017). Under natural conditions, this behavior is inherently risky.

Here, we examined whether activity in the open‐field changes with season, body mass, and reproductive condition in a wild rodent, the yellow‐necked mouse *Apodemus flavicollis*. We tested the following predictions:

1. Exploration will be higher in the spring than in the fall. During spring, when reproductive season starts, individuals should maximize resource intake to increase reproduction. In the fall, reproduction will not happen until next spring. This should promote investment in survival (“asset‐protection”), and more cautious behavior than in the spring.
2. Exploration will be positively associated with body mass. Small, juvenile individuals must survive before they can reproduce, thus should be more cautious (invest in future reproduction) than medium‐sized individuals (which should invest in both current and future reproduction), and especially than large adults, which are expected to invest mostly in current breeding (Clark, 1994). However, this is not the only relationship that is suggested by theory. Alternatively (2a), exploration will be negatively associated with body mass. Body mass scales with age, and aging individuals receive lower marginal benefits from information gathered through exploration (Sherratt & Morand‐Ferron, 2018; Spreng & Turner, 2021). Thus, younger individuals should accept higher risk associated with exploring novel environments. Finally (2b), when the asset‐protection and the age‐related loss in the marginal benefits of information co‐occur, exploration will be U‐shaped (Sherratt & Morand‐Ferron, 2018), with high values in young and old individuals (as in Lafaille & Féron, 2014).
3. Exploration will be higher in reproductively active vs. non‐active individuals. Animals in breeding condition need to fuel the reproduction, thus have higher energy needs and should take more risks than non‐breeding individuals (Clark, 1994).
4. Exploration will be higher in males than in females. Males, who usually have higher potential reproductive rate and therefore more to gain (Glutton‐Brock & Vincent, 1991), are expected to be more willing to take risks in comparison with females (Palanza, 2001a).

In addition to testing these predictions, we also quantified repeatability of exploration in the open arena, adjusted for any significant effects of season, body mass, exploration, reproduction, and sex (Stoffel et al., 2017). In this manner, we assessed whether consistent inter‐individual differences in exploration remained even after accounting for the tested variables.

## 2 METHODS

### 2.1 Study species

The yellow‐necked mouse is a small, solitary nocturnal rodent from the Muridae family (Pucek, 1981). It is widely distributed in Europe and in western Asia, where it inhabits coniferous and deciduous forests. The yellow‐necked mouse is omnivorous, but specializes in tree seeds; therefore, it is particularly abundant in forest stands with nut‐bearing species such as beech and oak, and after years of intense seed production (Zwolak et al., 2016, 2018). Home ranges of females are smaller than those of males (Stradiotto et al., 2009) and remain relatively stable throughout the year; males greatly expand their home ranges in spring, when breeding starts, and compete by searching for receptive females (Montgomery, 1989). Male home ranges decrease again in autumn. The yellow‐necked mouse breeds between March and October, producing 2–4 litters per year (Pucek, 1981). Late‐born offspring becomes sexually mature after overwintering. Under natural conditions, the yellow‐necked mouse lives up to 1 ¼ years (Pucek, 1981), but typical lifespan is much shorter (3–4 months: Gasperini et al., 2016).

### 2.2 Study sites

The research was conducted in Gorzowska Forest, situated in western Poland (52.77°N, 15.07°E) at an altitude of 60–80 m. The Gorzowska Forest is located in the temperate climate zone, with average annual precipitation of 523 mm, average annual temperature of 8°C, and average monthly temperatures ranging from −4°C in January to 23°C in July (measured for the city of Gorzów Wielkopolski, 12 km from the study sites). The forest is managed for timber. Common tree species include European beech (*Fagus sylvatica*), oaks (*Quercus* spp.), Scots pine (*Pinus sylvestris*), and European larch (*Larix decidua*). The research was conducted in European beech stands.

### 2.3 Trapping

Rodents were trapped during 3 years: 2015 (in the fall), 2016 (spring and fall), and 2017 (spring and fall). The spring trapping was conducted in April and May. The fall trapping was conducted in September and October. Typically, we conducted one trapping session per site per month, and the trapping session lasted for 5 consecutive days and nights. However, there were exceptions from this pattern due to disturbance by animals (wild boars *Sus scrofa* and raccoons *Procyon lotor* overturning traps), with some trapping sessions shortened or cancelled.

In total, we established nine trapping grids. Two of them were used in 2015, eight were used in 2016 and 2017. Each grid consisted of 64 wooden live‐traps spaced 10 m apart, creating a 0.49 ha square. The traps (widely used in Poland “dziekanówka” type, 21 × 8 × 9.5 cm; produced by PPUH A. Marcinkiewicz, Rajgród, Poland) were baited with rolled oats and sunflower seeds. We checked traps in the morning (starting at 8 am) and in the evening (starting at 6 pm). We identified captured rodents to species, determined their sex and reproductive status, measured their body mass to the nearest 0.5 g with Pesola™ spring balances, and marked them with uniquely numbered ear tags (National Band and Tag Company, Newport, USA) and with PIT tags (Biomark HPT 12 PIT Tag). Tagging was always conducted after behavioral tests, and animals were released immediately after the procedure. The reproductive status was denoted as “active” when males were scrotal, and females were pregnant, lactating, or had an open vagina. The status was “inactive” when males were non‐scrotal, and females had a closed vagina and did not exhibit signs of pregnancy (visibly distended abdomen) or lactation (nipples with signs of sucking). Besides yellow‐necked mice, we captured bank voles (*Myodes glareolus*) and occasionally striped field mice (*Apodemus agrarius*), Eurasian harvest mice (*Micromys minutus*), field voles (*Microtus agrestis*), Eurasian common shrews (*Sorex araneus*), and Eurasian pygmy shrews (*S*. *minutus*).

### 2.4 Behavioral tests

**FIGURE 1:**
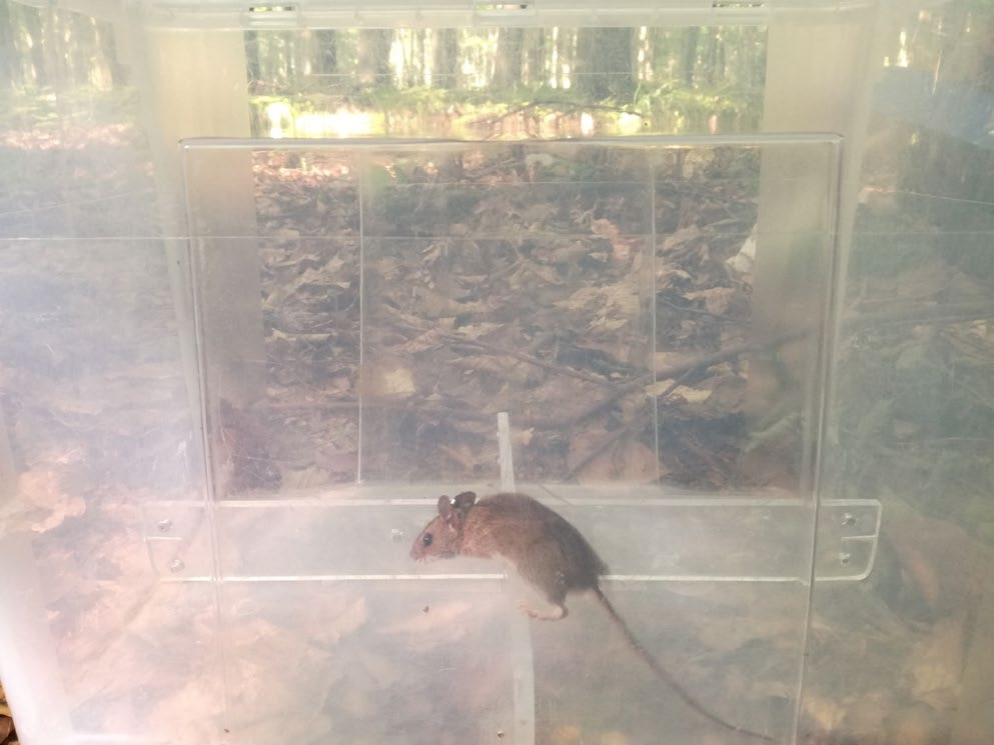
A yellow‐necked mouse during the open‐field test (photo by Paulina Celebias)

Upon capture, yellow‐necked mice were tested on the study site for behavioral types. We measured behavioral responses to a novel environment with an “open‐field” type of test (Archer, 1973; Carter et al., 2013; Montiglio et al., 2010; Perals et al., 2017). Captured individuals were carried in traps to the open‐field arena, located by the trapping grid. Then, they were transferred from traps to cotton bags and gently placed in the testing arena. The arenas were made of 28 × 40 × 34 cm plexiglass boxes. Floors of the boxes were divided into four sections with two low (4.5 cm), perpendicular partitions (Figure 1). Behavior of tested mice was recorded with hand‐held cameras for 2 min, while the observers remained silent and still (see e.g., Bergeron et al., 2013 or Montiglio et al., 2014 for a similar procedure). Animals widely differed in their response to the novel environment. Some individuals froze and remained motionless throughout the test. Others slowly explored the new environment. Finally, some animals were highly active throughout the test. We recorded the number of times mice crossed the partitions (a measure of exploration: Réale et al., 2007). We note that the behavior of animals was recorded in short tests, immediately after placing in the new environment. The capacity to explore under such (most likely stressful) conditions can also be interpreted as “boldness”. However, as a rule of thumb, boldness should be measured in a familiar environment and exploration in a novel one, such as the open arena in our experiments (Réale et al., 2007). At any rate, exploration and boldness are often correlated (also genetically: van Oers et al., 2004), and, crucially for our inferences, both represent risky behaviors (Réale et al., 2007).

After each test, the arena was thoroughly wiped with 70% alcohol. To standardize conditions, the tests were conducted only after the morning trapping sessions and only during non‐rainy days (because the sound of rain drops hitting the box distracted the animals). Females in advanced stages of pregnancy and females with signs of intense lactation were released without testing. All trapping and testing protocols were conducted under approval (no. 54/2015, issued on 03.07.2015) of the Local Ethical Commission for Experiments with Animals in Poznań (Poland).

### 2.5 Statistical analyses

The analyses were conducted in R (R Core Team, 2018) using package *glmmTMB* (Brooks et al., 2017) for model fitting and *rptR* (Stoffel et al., 2017) for calculating repeatability. We compared body masses between sexes, seasons, and reproductive states with t‐tests. We tested all predictions by constructing generalized linear mixed models (GLMMs) with the number of crossings as the response variable. Explanatory variables included “season” (spring vs. fall), “body mass” (both linear and quadratic effect), “sex” (male vs. female), and “reproductive status” (active vs. inactive). We also included two interactions, “body mass * season” and “reproductive status * season”, to account for the possibility of contrasting seasonal strategies in animals with different age or reproductive status. In addition to these variables, which were directly related to our predictions, we included also nuisance explanatory variable such as “year” (because the tests were conducted over 3 years), and “test number” (because repeatedly tested individuals often show signs of habituation: Finger et al., 2016; Underhill et al., 2021). “Individual” (i.e., mouse PIT tag number) and “trapping plot” were entered as random effects. We used the negative binomial error family with zero‐inflation (detected with package *performance*: Lüdecke et al., 2021).

We calculated adjusted repeatability of the open‐field test results using GLMMs fitted with package *rptR* (Stoffel et al., 2017). We included “individual” as the random effect and controlled for fixed effects that were found significant in the GLMM analysis above. We did not exclude individuals with a single test because censoring such individuals reduces, rather than improves, power in random regressions (Martin et al., 2011). We used the Poisson error family, and performed 1000 parametric bootstrap iterations. We present the results on both the original and the link scale (Stoffel et al., 2017).

The main analysis was supplemented by several additional, post‐hoc analyses that are presented in Appendix. We tested whether the results are influenced by the unequal abundances of individuals with different body masses (Table A1), and by the presence of pregnant females (Table A2); we also examined how the outcomes change when we divide individuals into light (juveniles) and heavy (adults) instead of using body mass as a continuous variable (Table A3), when we include year*season interaction (Table A4), when we limit the dataset to only one open‐field test per individual (Table A5), and when we include sex * season interaction (Table A6).

## 3 RESULTS

**FIGURE 2:**
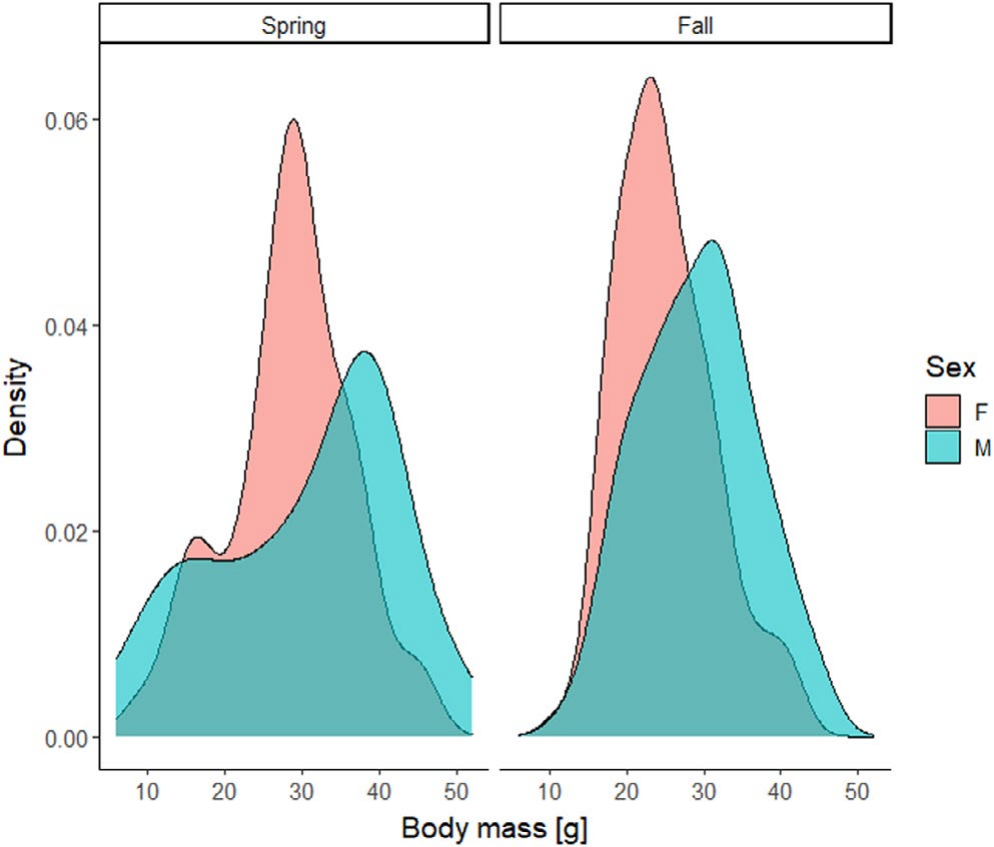
Body mass of male and female yellow‐necked mice (*Apodemus flavicollis*) captured during spring and fall trapping

In total, we tested 273 individuals (151 males and 122 females), with an average of 1.7 tests per individual (range: 1–8). One hundred and seven individuals were tested in the spring and 176 in the fall (10 individuals were tested in both seasons). In the spring, 53% of individuals were reproductively active; in the fall, this proportion equaled 38%. The average body mass was slightly higher in the spring than in the fall (29.6 g vs. 27.3 g, *p* = .04), in males than in females (29.8 g vs. 26.3 g, *p* < .001; Figure 2), and in reproductively active vs. non‐active individuals (32.6 vs. 24.5 g, *p* < .001).

**FIGURE 3:**
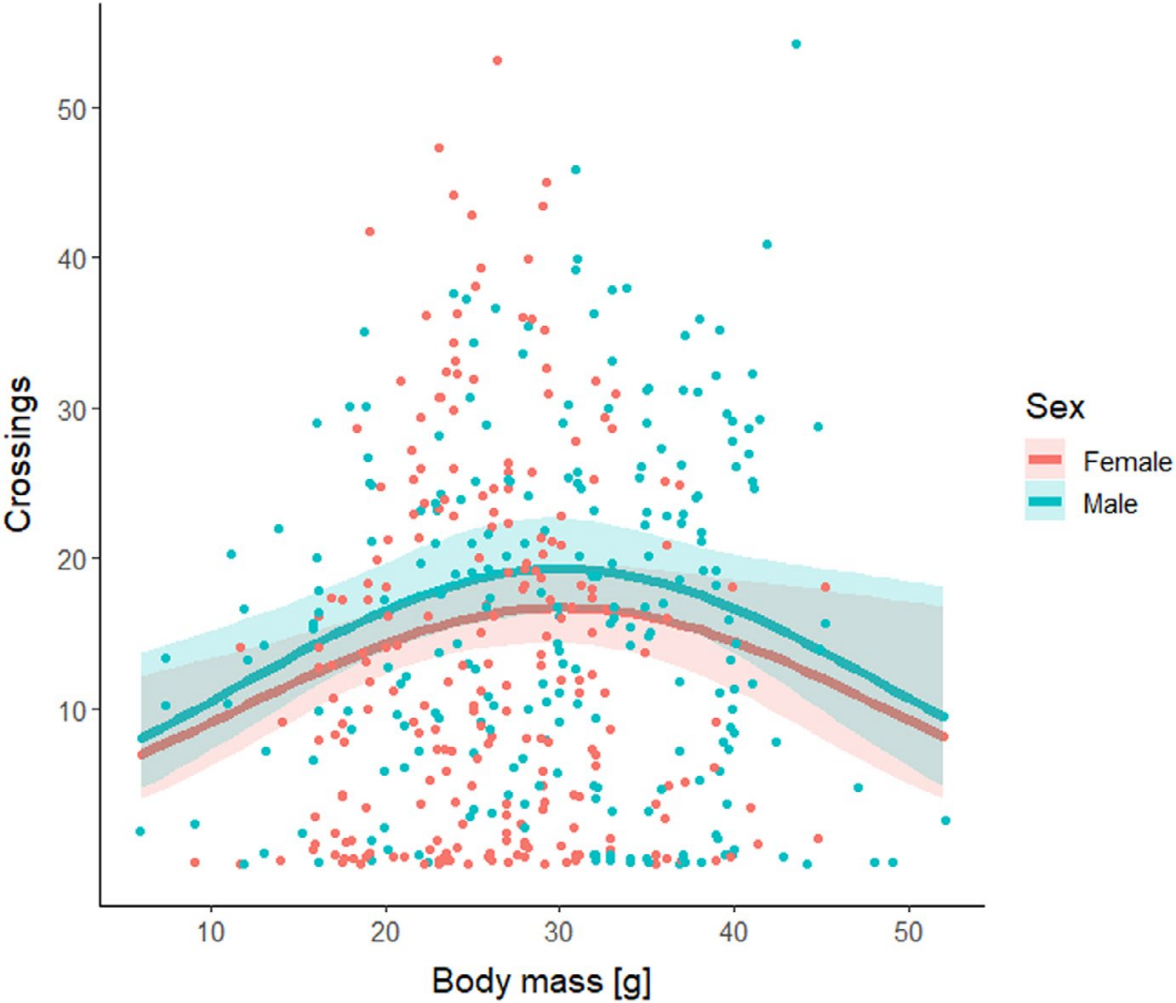
A relationship between body mass and partition crossings in the open‐field tests, for male and female yellow‐necked mice (*Apodemus flavicollis*). Dots indicate data points (jittered to improve visibility), lines represent estimated averages, and shading corresponds to 95% confidence intervals. See Table 1 for model summary

Contrary to prediction (1), exploration in the open field was not associated with season (Table 1). Post‐hoc analyses indicated that the effect of season on exploration possibly varied among years (Table A4). Contrary to predictions (2) and (2a), it did not change linearly with body mass. Instead, the relationship between exploration and body mass was hump‐shaped, but contrary to prediction (2b) exploration was highest in animals with medium body mass (Table 1, Figure 3). Contrary to prediction (3), the number of crossings was not influenced by reproductive status (Table 1). In agreement with prediction (4), males tended to score slightly higher than females (Table 1, Figure 3). Nuisance variables “year” and “test number” did not affect the number of crossings (Table 1). The quadratic effect of body mass retained its significance in all, and sex effect in most additional, exploratory models (Tables A1, A2, A3, A4, A5, A6).

**TABLE 1:** Summary of the model output that tested the relationship between exploration in the open field (“Crossings”) and the following variables: year (2015, 2016, and 2017), season (spring and fall), body mass (standardized by *z*‐scoring), sex (female and male), reproductive state (active vs. inactive), and test number (1–8 tests per individual). The exploration was measured by the number of partition crossings during a 2‐min test. Significant results are in bold. The model is a zero‐inflated negative binomial GLMM (generalized linear mixed model) – see “Methods” for details

Predictors	Crossings		
	Chi‐square	*df*	*p*
Year	1.012	2	.603
Body mass (quadratic)	7.365	1	**.007**
Season	0.738	1	.390
Sex	4.159	1	**.041**
Reproductive state	2.238	1	.135
Test number	2.111	1	.146
Body mass (quadratic) * season	0.003	1	.954
Season * reproductive state	0.926	1	.336
Observations	460		

In addition to being influenced by body mass and sex, the exploration scores showed considerable between‐individual variation that was consistent in the repeated tests. The number of crossings in the open‐field tests had adjusted repeatability (i.e., after taking into account the effects of body mass and sex) of 0.52 on the link‐scale (95% CI: 0.39–0.62, *p* < .001) and 0.37 on the original scale (95% CI: 0.26–0.47, *p* < .001).

## 4 DISCUSSION

Despite our sample size, which was relatively large for behavioral data, we did not find an association between exploration in the open‐field and season or reproductive status. Furthermore, the relationship between exploration and body mass did not fit any of our a priori predictions. On the other hand, the results supported the prediction that males will have higher exploration than females, even though the effect size was rather small.

Individuals experience shifts in social and environmental conditions as seasons change. Hypothetically, these shifts could trigger expression of different life‐history strategies (Eccard & Herde, 2013). However, the lack of consistent association between season and exploration in our study differs from results on common voles (*Microtus arvalis*), which were more active in open‐field tests in spring relatively to other seasons (Eccard & Herde, 2013). Similarly, in coho salmon (*Oncorhynchus kisutch*), the willingness to take risk declined from spring to autumn (Reinhardt & Healey, 1999), and warblers *Sylvia borin* and *S*. *melanocephala* were most likely to investigate novel objects in spring (Mettke‐Hofmann, 2007). However, the predictions on risk taking and season that originate from the asset‐protection principle are highly susceptible to changes of assumptions (e.g., the timing of reproduction or the existence of metabolic constraints), and in some cases incorporating details on species‐specific biology can fundamentally change the expected relationship (Grand, 1999; Reinhardt, 2002). This can explain varied results from other studies (e.g., Greggor et al., 2016; Magnhagen & Vestergaard, 1991; Uchida et al., 2016).

In principle, the apparent lack of relationship between season and exploration could also result from contrasting seasonal strategies in animals with different age or reproductive status. We did not age animals, but distribution of body masses in the spring suggests that the studied populations contained a mixture of young‐of‐the‐year and overwintered animals (Figure 2). Old, overwintered animals are expected to have a low residual reproductive value, thus should be risk prone. Young‐of‐the‐year animals are expected to have a high residual reproductive value, thus should be risk averse (Clark, 1994). However, if these two classes of animals differed in season‐dependent exploration strategies, there should be an interaction between body mass and season. None was found (Table 1).

Similarly, our fall sample, in addition to non‐breeding animals, contained individuals that were reproductively active. This again could lead to contrasting exploration strategies because breeding individuals should accept more risk to capitalize on reproduction before the end of breeding season (Clark, 1994). Thus, the predicted autumn decline in exploration would be apparent only in non‐breeding animals. Yet, again, there was no interaction between season and reproductive state (Table 1).

Additional analyses suggested that the direction of seasonal changes in exploration changed from year to year (Table A4). This could reflect the influence of unmeasured environmental variables on rodent behavior. Yet, the interaction between season and year was only marginally significant, and conducting post‐hoc analyses inflates the risk of false‐positive discoveries (Forstmeier et al., 2017). Thus, this effect should be verified in future studies.

Body mass had a quadratic association with exploration in the open field. Animals with the intermediate mass were the most explorative. This does not fit the relationships predicted by the asset‐protection principle or the age‐related shift hypothesis (Clark, 1994; Sherratt & Morand‐Ferron, 2018). We note, however, that the asset protection can generate a variety of predictions that depend on details of relationships between body mass, age, and reproduction (Clark, 1994; Reinhardt & Healey, 1999; Sherratt & Morand‐Ferron, 2018).

As a caveat, body mass confounds individual's age and condition. Advanced age reduces reproductive value, but good body condition might increase it (Clark, 1994). We did not attempt to calculate a separate body condition index (e.g., Labocha et al., 2014; Peig & Green, 2010) because in our experience, measurement error makes such indices unreliable when working with living, wild rodents under field conditions (see also Krebs & Singleton, 1993 for a detailed discussion of this problem). However, the effects of age on body mass are likely to overwhelm the effects of body condition. Mice weigh less than 10 g when they leave the nest and old adults can weigh more than 60 g (Pucek, 1981). The effects of body condition on body mass are likely to be much more modest. Moreover, in animals that cache food (such as *A*. *flavicollis*: Zwolak et al., 2018, Bogdziewicz et al., 2020), the importance of body reserves is reduced and the relationship between body mass and individual's condition is weakened (Underhill et al., 2021).

The hump‐shaped relationship between body mass and exploration that was found in this study most closely resembles patterns reported in house mice *Mus musculus* (Macrì et al., 2002). Subadult house mice (which had intermediate body mass) showed higher levels of exploration in the open field than both juveniles (which were relatively lighter) and adults (relatively heavier). This pattern was explained by a strong drive to disperse in the subadult mice, which is related to rodent life history, i.e., dispersal of adolescent individuals from natal home ranges. During this period, risk taking and exploration of novel environments might be selectively favored (Macrì et al., 2002). In support of this line of reasoning, subadults are overrepresented among dispersers in the yellow‐necked mouse (Gliwicz, 1988; Rajska‐Jurgiel & Mazurkiewicz, 2000). We note, however, that in our study, the highest exploration occurred in animals that weighed almost 30 g, whereas dispersers in field studies of yellow‐necked mice typically weigh only about 20 g (Gliwicz, 1988; Rajska‐Jurgiel & Mazurkiewicz, 2000).

There was no association between the reproductive status of yellow‐necked mice and their exploration level. This lack of effect contrasts with theoretical predictions (Clark, 1994) and certain empirical studies (e.g., Greggor et al., 2016). However, yet again, the existence and direction of the link between reproductive activity and risk taking depends on specific aspects of the reproductive effort and predation risk. For example, breeding animals should be less responsive to predation risk when the danger is chronic, but when the danger is sporadic, cautious behavior is advantageous even during breeding (Clark, 1994).

Males exhibited a higher exploration in the open field than females. This result conforms to theoretical expectations of higher risk taking in males than in females (Palanza, 2001a). Such a pattern is well documented in humans, where across cultures men are more risk prone when compared to women (Apicella et al., 2017; Archer, 2019). However, sex differences in risky behavior appear highly species‐ and context‐specific (e.g., Blaszczyk, 2017; Habig et al., 2017; Krenhardt et al., 2021). Also in rodents, behavioral tests on sex‐related differences in risk taking and exploration in rodents brought mixed results (Gore‐Langton et al., 2021; Montiglio et al., 2010; Orsini et al., 2016; Palanza, 2001a; Palanza et al., 2001b; Vošlajerová Bímová et al., 2016). This variation precludes broad empirical generalizations on the link between sex and risk taking. Clearly, there is a need for a new predictive framework to explain and organize these differences. For example, the existence or lack of male–female differences in exploration tendency could depend on the mating system (scramble competition for mates, which occur in the yellow‐necked mouse, can promote increased exploration tendency in males: Jašarević et al., 2012).

Lastly, the variation in exploration scores had a considerable among‐individual component. It was consistent in repeated tests; thus, it fulfilled the definition of personalities (differences across individuals that are consistent within individuals over time: Stamps & Groothuis, 2010; see also Sih et al., 2004 and Réale et al., 2007 for similar definitions). Repeatability of the open‐field exploration scores in this study was comparable to typical values reported in animal personality studies (0.37, according to a meta‐analysis by Bell et al., 2009). High repeatability is more likely to be found when the interval between repeated tests is short (Bell et al., 2009). Even though we avoided testing on subsequent days, most tests on the same individuals were conducted during a single trapping session (i.e., within 5 days). However, small mammal populations have a very high turnover. Individuals typically live only a couple of months (e.g., Gasperini et al., 2016; Supp et al., 2015), thus, in small mammals, short‐term repeatability matters.

## 5 CONCLUSIONS

Patterns of open‐field exploration found in this study did not support predictions based on the asset‐protection principle. However, the asset protection can take many forms that are contingent on specific aspects of the relationships between rewards (increased resource acquisition), costs (increased mortality), and risk taking. Depending on assumptions, the connection between risk taking and factors that (putatively) influence reproductive value varies. Even one of the most fundamental, and seemingly obvious assumptions, i.e., that risky behavior reduces survival, can depend on context. As a prominent example, a recent meta‐analysis suggested that in the wild, individuals expressing greater risky behaviors live longer (Moiron et al., 2020). While contrary to predictions, our results on exploration vs. body mass might be linked to age‐specific patterns of exploration and dispersal in rodents (Gliwicz, 1988; Macrì et al., 2002; Rajska‐Jurgiel & Mazurkiewicz, 2000), and the influence of sex on exploration in the open field conforms to long‐standing theoretical notions (Palanza, 2001a). Finally, the presence of strong and highly consistent interindividual differences in exploration tendency suggests the existence of individuals strategies (“behavioral types”) that go beyond the effects of body mass and sex.

## CONFLICT OF INTEREST

The authors declare no conflict of interest.

## AUTHOR CONTRIBUTIONS

**Paula A. Bednarz:** Investigation (lead); Writing – review & editing (lead). **Rafał Zwolak:** Formal analysis (lead); Funding acquisition (lead); Writing – original draft (lead).

## Data Availability

Data are available in the Knowledge Network for Biocomplexity (https://knb.ecoinformatics.org) at https://doi.org/10.5063/F1HQ3XCK.

## 1

**TABLE A1:** The hump‐shaped relationship between body mass and exploration in the open field was not driven by higher abundance of individuals with intermediate body mass

Predictors	Crossings		
	Chi‐square	*df*	*p*
(A) Dataset 1 (286 observations)			
Year	2.59	2	.274
Body mass (quadratic)	8.82	1	.**003**
Season	0.07	1	.792
Sex	8.28	1	.**004**
Reproductive state	7.08	1	.**008**
Test number	0.87	1	.351
Body mass (quadratic) * season	0.37	1	.544
Season * reproductive state	0.55	1	.459
(B) Dataset 2 (285 observations)			
Year	0.08	2	.961
Body mass (quadratic)	5.88	1	.**015**
Season	0.68	1	.410
Sex	4.09	1	.**043**
Reproductive state	2.09	1	.159
Test number	1.63	1	.201
Body mass (quadratic) * season	>0.01	1	.948
Season * reproductive state	1.07	1	.300

Note

We randomly assigned all records with intermediate body mass (i.e., between 20 and 40 g; 349 out of 460 records) to groups “1” and “2”. Then we removed either group 1 or 2 from the dataset and repeated our analyses with sample size for mice with intermediate body masses reduced by half. Significant effects are in bold. The relationship between body mass and exploration (crossings) remained robust. Other main results were also unchanged, although in Dataset 1 “reproductive state” became significant, most likely due to chance.

**TABLE A2:** Removing pregnant females from the dataset did not change our main results

Predictors	Crossings		
	Chi‐square	*df*	*p*
Year	1.41	2	.495
Body mass (quadratic)	4.19	1	.**041**
Season	1.32	1	.250
Sex	3.98	1	.**046**
Reproductive state	1.16	1	.282
Test number	3.68	1	.055
Body mass (quadratic) * season	0.21	1	.650
Season * reproductive state	0.48	1	.487
Observations	415		

Note

Significant effects are in bold. All variables that are relevant to our hypotheses retained their significance (or non‐significance).

**TABLE A3:** Dividing individuals into juveniles (≤20 g) and adults (>20 g) did not lead to new insights

Predictors	Crossings		
	Chi‐square	*df*	*p*
Year	1.07	2	.587
Age	5.59	1	.**018**
Season	2.12	1	.145
Sex	1.90	1	.168
Reproductive state	0.31	1	.578
Test number	1.78	1	.183
Age * season	1.32	1	.250
Season * reproductive state	0.61	1	.436
Observations	460		

Note

Significant effects are in bold. Animals lighter than 20 scored lower on exploration, as expected from patterns on Figure 1 in the main text. Division of animals into two categories of body mass masked the effect of sex on exploration scores. This model has considerably less support than the model with quadratic effect of weight (delta AIC = 6.1).

**TABLE A4:** The influence of season differed between years 2016 and 2017

Predictors	Crossings		
	Chi‐square	*df*	*p*
Year	0.87	2	.351
Body mass (quadratic)	3.99	1	.**046**
Season	0.66	1	.415
Sex	3.75	1	.053
Reproductive state	3.32	1	.068
Test number	1.51	1	.219
Year * Season	4.38	1	.**036**
Body mass (quadratic) * season	0.02	1	.896
Season * reproductive state	0.59	1	.443
Observations	396		

Note

In this analysis, sample size was reduced because to include year*season interaction, we excluded data collected in 2015, when sampling was conducted only in the fall. Significant effects are in bold.

**TABLE A5:** When the dataset was limited to only one open‐field test per individual, the effect of body mass, but not sex, retained its significance

Predictors	Crossings		
	Chi‐square	*df*	*p*
Year	3.73	2	.155
Body mass (quadratic)	5.16	1	.**023**
Season	0.54	1	.462
Sex	1.49	1	.222
Reproductive state	0.40	1	.528
Observations	273		

Note

As a caveat, this model discards all information gathered from repeated tests of the same individuals. The model is limited to main effects because including interactions lead to convergence problems.

**TABLE A6:** Including sex * season interaction did not change the main results

Predictors	Crossings		
	Chi‐square	*df*	*p*
Year	1.01	2	.602
Body mass (quadratic)	6.91	1	.**009**
Season	0.75	1	.388
Sex	4.19	1	.**041**
Reproductive state	2.23	1	.135
Test number	2.10	1	.148
Body mass (quadratic) * season	>0.01	1	.983
Season * reproductive state	0.92	1	.338
Season * sex	0.01	1	.922
Observations	415		

Note

All variables that are relevant to our hypotheses retained their significance (or non‐significance).
